# A moving story: Whole-body motor training selectively improves the appraisal of action meanings in naturalistic narratives

**DOI:** 10.1038/s41598-017-12928-w

**Published:** 2017-10-02

**Authors:** Piergiorgio Trevisan, Lucas Sedeño, Agustina Birba, Agustín Ibáñez, Adolfo M. García

**Affiliations:** 1 Department of Languages and Literatures, Communication, Education and Society, University of Udine, via Petracco 8, 33100 Udine, Italy; 2 Laboratory of Experimental Psychology and Neuroscience (LPEN), Institute of Cognitive and Translational Neuroscience (INCYT), INECO Foundation, Favaloro University, Pacheco de Melo 1860, C1126AAB Buenos Aires, Argentina; 3 National Scientific and Technical Research Council (CONICET), Av. Rivadavia 1917, C1033AAJ Buenos Aires, Argentina; 4 Universidad Autónoma del Caribe, Calle 90, N° 46-112, C2754 Barranquilla, Colombia; 5 Center for Social and Cognitive Neuroscience (CSCN), School of Psychology, Universidad Adolfo Ibáñez, Diagonal Las Torres, 2640 Santiago de Chile, Chile; 6 Centre of Excellence in Cognition and its Disorders, Australian Research Council (ARC), Sydney, Australia; 7 Faculty of Education, National University of Cuyo (UNCuyo), Sobremonte 74, C5500 Mendoza, Argentina

## Abstract

This study examined whether systematic whole-body stimulation and increased attention to visuospatial motion patterns can enhance the appraisal of action meanings evoked by naturalistic texts. Participants listened to action and neutral (non-action) narratives before and after videogame-based bodily training, and responded to questions on information realized by verbs (denoting abstract and action processes) and circumstances (conveying locative or temporal details, for example). Strategically, we worked with dyslexic children, whose potential comprehension deficits could give room to post-training improvements. Results showed a selective boost in understanding of action information, even when controlling for baseline performance. Also, this effect proved uninfluenced by short-term memory skills, and it was absent when training relied on non-action videogames requiring minimal bodily engagement. Of note, the movements described in the texts did not match those performed by participants, suggesting that well-established effector- and direction-specific language embodiment effects may be accompanied by more coarse-grained sensorimotor resonance, driven by activation of motor and visuospatial sensory systems. In sum, the stimulation of movement-related mechanisms seems to selectively boost the appraisal of actions evoked by naturalistic texts. By demonstrating such links between two real-life activities, our study offers an empirical tie between embodied and situated accounts of cognition.

## Introduction

In line with the embodied cognition framework^1^, several studies show that action training can causally impact the understanding of words and sentences denoting bodily movements^2^. However, two critical issues remain unresolved: (i) could such effects extend beyond atomistic tasks and emerge in naturalistic text processing?; and (ii) can they be triggered by immersive, whole-body training activities engaging motor and motion-related sensory systems? To address both questions, we implemented a videogame-based motor training protocol and assessed the appraisal of actions in narratives through discourse-level processes subsuming multiple operations (e.g., word access, syntactic parsing, semantic integration, memory retrieval). Strategically, our intervention was aimed at dyslexic children, whose potential comprehension deficits^3,4^ could give room to post-training improvements. As in previous research on text-level action appraisal^5^, our approach allows examining whether embodied language mechanisms remain operative despite the conflation of processes typical of daily verbal activity.

Processing of action-related language can be influenced by physical behaviors and motor-training experiences^2,6^. For instance, sustained practice of specific manual skills, such as bean displacement and origami, can differentially affect comprehension of directionally compatible action sentences^7^ and movement-congruent sentence-picture pairs^8^, respectively. While these studies indicate that motor training can specifically modulate action-language processing, they restrict post-training assessment to isolated, randomly sequenced verbal stimuli –for a review, see Yang^2^. However, motor networks also ground action language during discourse-level processing. Faced with real narratives, readers exhibit modulations in action-related regions as a function of noun manipulability^9^ and increased somatosensory cortex activity when characters change their interactions with an object^10^. Also, motor network damage decreases reliance on action-related semantic fields during spontaneous monologues^11^. By jointly considering both strands of evidence, we reasoned that motor training could distinctively boost the appraisal of actions evoked by naturalistic texts.

Regarding our second question, note that training protocols to date have employed highly artificial motor tasks which rarely, if ever, occur outside laboratory settings. Indeed, experiments based on bean displacement^7^ or deliberate rehearsal of pre-determined manual actions^8^, for example, fail to engage the entire body in a contextually immersive way. Yet, field-specific action-language understanding can be boosted by naturalistic motor expertise. For instance, professional athletes exhibit advantages in processing discipline-specific action verbs^12^, with accompanying increases of neural activity^13^ and connectivity^14^ in motor/premotor regions. Complementarily, increased attention to visuospatial motion patterns during non-manipulated whole-body action videogame (AVG) playing can improve the efficiency of the magnocellular dorsal pathway or action stream^15^. This warrants the conjecture that training protocols based on daily life activities could also hone motion-related mechanisms and favor the appraisal of action-related meanings –crucially including, but not limited to, their comprehension.

In sum, we hypothesized that the appraisal of actions in naturalistic narratives could be distinctively enhanced by systematic practice of a real-life bodily activity. To test this notion, we assessed processing of information in two types of short stories –“action texts” (ATs) and “neutral texts” (NTs)– before and after motor training. We targeted dyslexic children, a population with intact motor abilities, potentially suboptimal comprehension skills^3,4^, and susceptibility to videogame-induced effects^15^. Our training protocol required participants to play whole-body AVGs on a Nintendo Wii, which allowed us to circumvent some of the artificial constraints characterizing classical paradigms in cognitive science (such as the continuous performance of a single motor action in the context of an otherwise static body). Importantly, these games elicit multiple patterns of simultaneous and coordinated multi-limb movements, and they can induce specific cognitive enhancements in dyslexic children^15^.

Moreover, to explore whether potential effects were specific to AVGs, we conducted a complementary study on a separate mini-sample of dyslexic children using non-action videogames. Finally, given that videogame training boosts phonological short-term memory in dyslexic children^16^, we analyzed the role of this domain in our hypothesized effects. This way, we assessed whether systematic, immersive physical activity can selectively improve the appraisal of actions evoked by naturalistic narratives.

## Methods

### Participants

Twenty-two English-speaking Australian children were recruited following four criteria, all verified by their parents upon signing the study’s consent form: (i) confirmed diagnosis of dyslexia, (ii) no history of psychiatric or neurological disease, (iii) minimal exposure to videogames in the last six months, and (iv) commitment not to play videogames at home during the course of the study. However, two of them failed to complete all sessions in the protocol and were thus removed from the study. The final sample (*n* = 20) comprised 13 males and 7 females, with a mean age of 9.8 years (*SD* = 1.4) and an average of 3.5 years of education (*SD* = 1.5). All but three children were right-handed, and none of them had visual, auditory, or motor impairments. An estimation analysis showed that this sample size conferred sufficient statistical power to reach reliable effects (see Supplementary Material).

All participants were diagnosed as dyslexic by psychologists or speech therapists. Formal assessments corroborated such diagnoses, indicating mild difficulties in relevant tasks. The sample exhibited borderline performance in word reading (percentile of the sample’s mean = 18.50) and phonemic decoding (percentile of the sample’s mean = 26.82), as assessed through the Sight Words and Phonemic Decoding subtests of the TOWRE 2^17^. In both subtests, the sample fell in the lowest 30% of the Australian population, based on age-specific norms^18^. However, none of the children attended special classes. Importantly, although most of them had regular speech therapy sessions outside school, no sessions were scheduled during the course of the study.

The mini-control-experiment involved 10 dyslexic English-speaking children, all of whom met the study’s inclusion criteria. The sample was matched for gender, age, years of education, and handedness with the main experiment’s group. For statistical details, see Supplementary Material, section ‘Participants’.

All participants’ parents gave written informed consent. The study was carried out in accordance with the principles of the Declaration of Helsinki and was approved by the Ethical Research Committee of Sydney University.

## Materials

### Texts

We created four simple short stories narrating a day in the life of different characters, ensuring their comparability across multiple syntactic, lexical, semantic, pragmatic, and discourse-level variables, as detailed below. In two of them, classified as ATs, the characters performed multiple bodily movements (more than 70% of their clauses involved action verbs, denoting physical activities). In the other two, classified as NTs, the characters mainly engaged in mental or affective processes (more than 80% of their clauses revolved around existential, mental, and relational verbs implying no physical action). The classification of verbs was made by two expert linguists (PT and AMG) following semantic, syntactic, and distributional criteria described by Halliday and Matthiessen^19^. One text of each class was used for pre-training assessment, and the other two were used for post-training assessment.

To maximize structural comparability among the texts, we constructed them by first formulating eight syntactic patterns based on systemic-functional categories^19^, which were then filled with specific lexical items. For example, one of the patterns was “Clause complex: clauses in paratactic relation (extension: addition)”. This pattern was filled as “He gave him the money and added some more coins” (i.e., [clause complex [clause 1: subject + action verb + complement(s)] [coordinator: ‘and’] [clause 2: elliptic co-referential subject + action verb + complement(s)]]) for one AT, and as “He was extremely happy and had many friends” (i.e., [clause complex [clause 1: subject + non-action verb + complement(s)] [coordinator: ‘and’] [clause 2: elliptic co-referential subject + non-action verb + complement(s)]]) for one NT. Thus, all texts had similar numbers of sentences, simple sentences, sentences with paratactic relations only, sentences with hypotactic relations only, sentences with both paratactic and hypotactic relations, and minor sentences. The corresponding grammatical patterns were pseudo-randomly distributed within each text to minimize the possibility of anticipation and priming effects in the second condition, and they were then filled with verbs satisfying the action vs. non-action opposition and additional words.

In addition to syntax, the four texts were carefully matched for several other relevant variables. First, they possessed very similar numbers of characters, words, nouns, adjectives, adverbs, and verbs. Second, the mean frequency, familiarity, syllabic length, orthographic length, and imageability of content words were statistically similar across the four texts – psycholinguistic data for all lexical items were extracted from N-Watch^20^. Third, data gleaned through an online survey completed by 15 adult readers indicated that the texts were matched for comprehensibility and coherence (on a 1-to-5 scale). Finally, we assessed the texts’ readability using two automatized indexes and confirmed that they had similar rankings (all four texts were ranked as fourth-grade level through the Powers-Sumner-Kearl Formula, and as third-grade via the Spache Readability Index). For full statistical details, see Table 1.

**Table 1:** Linguistic features of the action and neutral texts.

	Action Text 1	Neutral Text 1	Action Text 2	Neutral Text 2	*p-*value
Characters^a^	696	743	693	668	0.241^#^
Words	167	169	164	153	0.816^#^
Nouns	33	25	28	22	0.485^#^
Adjectives	6	14	5	14	0.058^#^
Adverbs	6	16	9	10	0.161^#^
**Verbs**	**32**	**32**	**32**	**31**	**0.999** ^**#**^
**Action verbs**	**25 (78.1%)**	**11 (34.4%)**	**25 (78.1%)**	**9 (29%)**	**0.004** ^**#**^
**Non-action verbs**	**7 (21.9%)**	**21 (65.6%)**	**7 (21.9%)**	**22 (71%)**	**0.002** ^**#**^
Mean content word frequency^b,c^	803	974.6	926.4	1014	0.857*
Mean content word familiarity^b,d^	593.2	582.4	598.6	598.3	0.419*
Mean content word imageability^b,e^	442.8	394.9	440.2	399.1	0.060*
Mean content word syllabic length^b^	1.3	1.5	1.3	1.4	0.156*
Mean content word orthographic length^b^	4.8	5.1	4.8	4.8	0.680*
Sentences	17	17	17	17	0.999^#^
Minor sentences	0	0	0	0	0.999^#^
Sentences with parataxis only	3	3	3	5	0.835^#^
Sentences with hypotaxis only	4	3	3	1	0.630^#^
Sentences with parataxis and hypotaxis	3	3	4	2	0.881^#^
Comprehensibility	3.9	3.6	3.25	4	0.619*
Coherence	3.7	3.6	2.75	4	0.471*
PSKF	4.4	4.55	4.22	4.39	—
SRI	3	2.8	3.5	2.7	—

*Notes*. PSKF: Powers-Sumner-Kearl Formula; SRI: Spache Readability Index (revised).

^a^Character count was performed without counting spaces.

^b^Psycholinguistic data was extracted from N-Watch (Davis, 2005), based on lemma counts.

^c^Frequency data was extracted from the CELEX written database, through N-Watch (Davis, 2005).

^d^Familiarity data was extracted from the MRC database, through N-Watch (Davis, 2005).

^e^Imageability data was extracted from the Bristol/MRC database, through N-Watch (Davis, 2005).

^#^ *p*-values calculated with chi-squared test.

^*^ *p*-values calculated with independent measures ANOVA.

The texts were audio-recorded by a native female speaker who spoke the same dialectal variety of English as the participants (Australian English). A smooth narration pace was used in all cases. The files were recorded in.mp3 format with stereo output, and they lasted roughly one minute each. The audio files in can be found in the Supplementary Material.

### Questionnaires

For each text, we designed a 16-question multiple-choice questionnaire. Half of the questions pointed to process-related information explicitly realized by verbs and verb phrases, and the other half aimed at circumstances (units denoting locative or temporal information, for example) explicitly realized by adverbial or prepositional phrases. For instance, the question “How did Donald go to his friend Tommy’s?” pointed to process-related information realized by a verb, while the question “Where did Donald look for his money?” pointed to circumstantial information realized by a prepositional phrase. All process-related questions in the AT questionnaires referred to action verbs, and all of those in the NT questionnaires pointed to non-action verbs. Thus, action-information questions had a contrastive condition within and across texts. Questions were presented following the order of the corresponding events in the texts, with a strict alternation between process-related and circumstantial questions.

Each question was accompanied by five options, namely: a correct response, a subtly incorrect response, a grossly incorrect response, a ridiculous response, and an ‘I don’t remember’ option. The order of these options was randomized across questions, except for the ‘I don’t remember’ option, which was always presented last. For example, the question “How did Donald go to his friend Tommy’s?” featured these options: “Driving (grossly incorrect)/Walking (subtly incorrect)/Running (correct)/Swimming (ridiculous)/I don’t remember”. Correct responses were given one point, while incorrect answers or the “I don’t remember” option were given zero points. Therefore, each questionnaire had a maximum score of 16 points (8 for process-related questions and 8 for questions about circumstantial information). The full questionnaires can be found in the Supplementary Material.

## Procedure

### Main experiment

**Figure 1:**
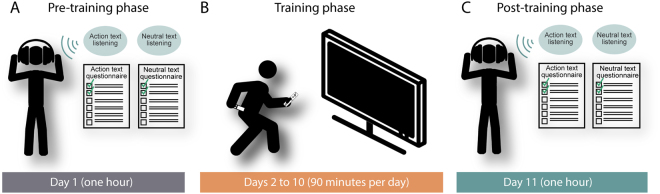
Study design. (**A**) Pre-training phase: on day 1, children listened to an action text and a neutral text, and, in each case, they answered a multiple-choice questionnaire tapping their appraisal of process-related and circumstantial information. (**B**) Training phase: from days 2 through 10, children completed a motor-training protocol based on action videogames. (**C**) Post-training phase: on day 11, children listened to another action text and another neutral text, and, in each case, they answered a multiple-choice questionnaire tapping their appraisal of process-related and circumstantial information.

The study comprised three phases: a pre-training (Pre-T) phase, a training (T) phase, and a post-training (Post-T) phase. In each of them, children performed the tasks individually under the supervision of an examiner. The structure of this longitudinal design is diagrammed in Fig. 1.

In the Pre-T phase, children listened to an AT and an NT through professional, high-definition headphones. Each text was played twice in a row. The order of presentation of the two text types (ATs vs. NTs), and the two texts within each text type (AT1 vs. AT2, and NT1 vs. NT2), was counterbalanced across participants. After each text, an examiner read the corresponding questionnaire out loud, repeating the questions and the options if necessary. The child chose an answer and the examiner marked it on the scoring sheet. Additional neuropsychological measures (i.e., a short-term memory task and the word reading and phonemic decoding measures reported in Section ‘Participants’) were administered upon completion of the text-based protocol.

The T phase consisted of nine sessions (one per day, through nine consecutive days) in which children played immersive, whole-body-active videogames on Nintendo Wii. All sessions were conducted in a customized laboratory at Sydney University, under supervision of an examiner. Each session lasted 90 minutes and was made up of two 40-minute gaming periods with a 10-minute break in between. The children played all games on their feet, standing at about 2 meters from a 23-inch Dell Optiplex 9030 AIO screen. They held a remote control on the right hand and a wireless nunchuck on the left hand, with which they performed various target-directed actions. Game audio was delivered through professional headphones.

The games were part of a collection titled Rayman Raving Rabbids, which has been used in previous videogame-based protocols^15^. These games require fast and precise bodily responses to successfully avoid or interact with moving objects and characters in transient events. Multiple action plans need to be adequately formulated and timely executed to achieve precision in target-aimed movements. The games elicited various types of bodily actions^16^ as children responded to temporally and spatially unpredictable events on the screen. In particular, both arms were constantly in motion (either individually or in coordination), as were the legs and the waist: in the “Shake your Booty” category (including 16 games), for example, children moved their bodies to the rhythm of dancing music while quickly moving the remote (in their right hand) and the nunchuck (in their left hand) when particular characters appeared on the stage. Each game was played for about 5 minutes and some of the games were repeated across sessions. All children played the same games for the same amount of time. Throughout the T phase parents made sure that children did not play other videogames outside the study sessions.

Finally, in the Post-T phase, the children listened to the AT and the NT which were not used in the Pre-T phase. The same neuropsychological measures used in the Pre-T phase were applied again between the texts. After listening to each text, the children were administered the corresponding questionnaires. The protocol for this phase was exactly the same as the one implemented for the Pre-T phase.

### Mini-control-experiment

To assess whether potential effects in our main experiment were due to video-gaming at large, as opposed to whole-body-active video-gaming in particular, we ran a mini-control-experiment involving minimally interactive, non-action videogames. This experiment involved 10 dyslexic English-speaking children who met the study’s inclusion criteria (see section ‘Participants’ above) and were sociodemographically matched with the main experiment’s sample (see Supplementary Material, section ‘Mini-control experiment Participants’).

The protocol was exactly the same as the one described in section ‘Procedure’, except that the games used during the T phase were characterized by a low degree of dynamic bodily engagement. These games, also included in the Rayman Raving Rabbids collection and used in previous studies^15^, are very mechanical and do not require ongoing adaptation to contextual stimuli. Most of them involved repeated responses from only one arm, and they did not elicit any movements from the legs, waist or upper body parts. Given their high degree of repetition, they could actually be successfully played with eyes closed (for details, see Supplementary Material, section ‘Non-action video games: further details’). As was the case in the main experiment, upon signing the study’s consent, parents ensured that the children did not play any other videogames throughout the T phase.

### Phonological short-term memory assessment

Using a previously reported phonological short-term memory task, we assessed whether potential training-induced enhancements in this domain^16^ influenced the predicted action appraisal effects. Participants listened to two lists (a ‘list couple’) containing an equal number of trigrams (nonwords made up of three sounds) and they were asked to repeat them. If at least one of the two lists in the couple was repeated correctly, a new ‘list couple’ was presented. The test included eight list couples (16 lists in total) and it ended as soon as the children made mistakes in the repetition of both lists. The span coincided with the last ‘list couple’ in which at least one of the two lists was repeated correctly. Therefore, the maximum possible score was 8.

### Statistical analysis

First, we performed a factorial ANOVA on text results, with a 2 × 2 × 2 design including the following factors: Text type (ATs and NTs), Information type (process related and circumstantial) and Time point (Pre-T and Post-T). Interaction effects were further scrutinized via Tukey’s HSD post-hoc tests. Then, to analyze the effect of training controlling for each participant’s baseline performance, we performed a factorial ANOVA on the subtraction between Post-T and Pre-T, using a 2 × 2 design with the factors Text type and Information type. Finally, to assess the impact of extra-linguistic skills on text appraisal results, significant differences were additionally assessed via an ANCOVA (using scores from the short-term memory task as a covariate). Effect sizes were calculated with Cohen’s *f* index.

No observations were excluded from either the main experiment or the mini-control-experiment. All independent variables considered and all dependent variables analyzed for both experiments have been reported in the Methods section. The datasets generated during and/or analyzed in this study are available from the corresponding author upon reasonable request.

## Results

### Main experiment

#### Text appraisal: ANOVA results

**Figure 2:**
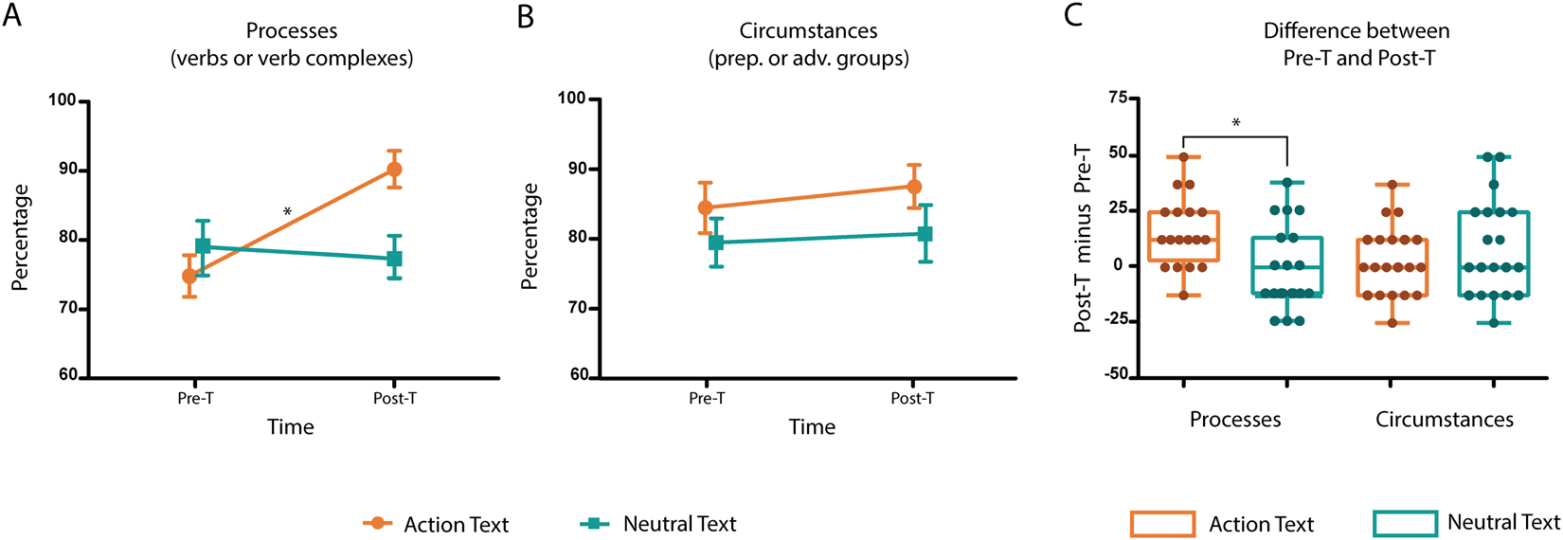
Text appraisal results from the main experiment. (**A**) The appraisal of process-related information (realized by verbs or verb groups) increased significantly only for action texts after AVG training. (**B**) The appraisal of circumstantial information (realized by prepositional or adverbial groups) was unaffected by AVG training in both action and neutral texts. (**C**) Subtraction analyses corroborated the finding that AVG training selectively enhanced the appraisal of process-related information for action texts. Values on the Y-axes indicate percentage scores. Asterisks (*) indicate significant differences. Pre-T: pre-test phase; Post-T: post-test phase.

We found a significant main effect of Text type [*F*(1, 152) = 9.25, *p* = 0.002, *f* = 0.55], with higher values for ATs than NTs. We also observed a main effect of Time point [*F*(1, 152) = 7.67, *p* = 0.006, *f* = 0.55], indicating higher scores in the Post-T than in the Pre-T phase. Crucially, the only significant interaction was among Text type, Information type, and Time point [*F*(1, 152) = 6.25, *p* = 0.013, *f* = 0.50]. Post-hoc comparisons, via Tukey HSD tests (MSE = 1.37, *df* = 152), revealed non-significant effects in Pre-T between ATs and NTs in process-related (*p* > 0.250) and circumstantial (*p* = 0.121) information, indicating that baseline performance was similar for all conditions. The only selective significant improvement emerged in the appraisal of process-related information for ATs (*p* = 0.017), with no comparable effect for NTs (*p* > 0.250) –Fig. 2, Panel A. Importantly, the appraisal of circumstantial information was not significantly affected by training, either in ATs (*p* > 0.250) or in NTs (*p* > 0.250) –Fig. 2, Panel B. Additionally, to better interpret the relevance of the triple interaction, we further assessed the improvement in the appraisal of process-related information for ATs with an ANOVA, revealing the specific effect size of this significant result. As expected, we found significant higher scores in the Post-T than in the Pre-T phase [*F*(1, 38) = 14.94, *p* < 0.001], with a large effect size (*f* = 1.27).

To further analyze the effect of training while controlling for the participants’ baseline performance in the Pre-T phase, we performed subtraction analyses between Post-T and Pre-T results. We found no main effects of Text type [*F*(1, 76) = 2.18, *p* = 0.145] or Information type [*F*(1, 76) = 0.5, *p* > 0.250]. The only significant effect emerged in the interaction between both variables [*F*(1, 76) = 8.21, *p* = 0.005, *f* = 0.70]. A post-hoc comparison via a Tukey HSD test (MSE = 2.08, *df* = 76) showed that training only induced a significant improvement in the appraisal of process-related information for ATs (*p* = 0.015). There was no significant effect on the comprehension of circumstantial information (*p* > 0.250) –Fig. 2, Panel C. As in the previous analysis, based on an ANOVA of the significant results of the process-related information, we found a large effect size of the differences between ATs and NTs: *F*(1, 38) = 10.59, *p* = 0.002, *f* = 1.06.

Importantly, the same results emerged upon replicating the analysis on 20 randomly selected subsamples of 10 participants each (the same *N* used in the mini-control-experiment). In 18 out of 20 of these analyses, we found a significant interaction between Text type, Information type, and Time point. Means (*m*) and standard deviations (*SD*) of this interaction’s statistics across the 20 analyses were *F*: *m* = 7.44, *SD* = 2.98; *p*-value: *m* = 0.020, *SD* = 0.030; *f*: *m* = 0.29, *SD* = 0.08 (for details of each model, see Table S1 in the Supplementary Material). This interaction was driven by a significant improvement in the appraisal of process-related information for ATs (*p*-value: *m* = 0.03, *SD* = 0.02), with no comparable effect for NTs (*p*-value > 0.250). Crucially, in these analyses, baseline performance was similar for all conditions, as results revealed non-significant effects in Pre-T between ATs and NTs in process-related and circumstantial information; also, training did not affect the appraisal of circumstantial information in ATs or NTs (for statistical details and illustration, see Supplementary Material, section ‘*Replication on reduced subsamples*’, Table S1, and Fig. S1). Taken together, such results suggest that our key finding was not merely driven by the fact that the main experiment’s sample was larger than that of the mini-control-experiment.

In sum, AVG training led to an improvement in the appraisal of text-level information, which was confined to process-related information in ATs (i.e., action verbs or verb phrases). Moreover, this selective effect remained the same even when controlling for differences in the subjects’ baseline performance in the Pre-T phase.

#### Short-term memory: ANOVA results

We compared the Pre-T and Post-T scores in the short-term memory task using an ANOVA. The difference between Pre-T (*M* = 2.4, *SD* = 1.23) and Post-T (*M* = 3.15, *SD* = 0.87) was significant [*F*(1, 38) = 4.93, *p* = 0.032, *f* = 0.73]. Thus, exposure to AVGs also induced enhancements in short-term memory.

#### Text appraisal: ANCOVA results

To assess the possible influence of short-term memory effects on the observed action appraisal enhancement, we reanalyzed the latter entering short-term memory performance as a covariate. Results indicated that the significant improvement in the appraisal of process-related information for ATs remained significant after covariation [*F*(1, 37) = 11.25, *p* = 0.002, *f* = 1.02]. We also reran the subtraction analysis between Post-T and Pre-T using short-term memory scores as a covariate. Once again, the selective enhancement for process-related information in ATs survived covariation [*F*(1, 37) = 11.25, *p* = 0.001, *f* = 1.10].

Taken together, these results suggest that the selective improvement in the appraisal of process-related information for ATs (i.e., action verbs and verb-phrases) was not influenced by short-term memory skills.

### Mini-control-experiment

We also analyzed results from the mini-control-experiment (in which participants were trained via non-action videogames). There were no main effects of Text type, Information type, or Time point, and the interaction among factors was also non-significant. The same was true for the subtraction analysis (see Fig. S2 in the Supplementary Material). No training-related effects were observed on short-term memory performance, either. For statistical details, see Supplementary Material, section ‘Results’.

Though speculative on account of the low sample size, these findings suggest that the selective action appraisal enhancement triggered by AVGs may not due to mere videogame exposure, but possibly associated with the engagement of motor and motion-related sensory systems proper.

## Discussion

After nine days of playing AVGs, participants selectively improved the appraisal of process-related information in ATs (i.e., action verbs and verb phrases). This pattern extends previous demonstrations that increased attention to visuospatial motion patterns distinctively engages the so-called action stream, enhancing performance in associated cognitive domains^15^. More particularly, our finding aligns with previous behavioral and neural stimulation studies evincing causal effects of motor-network entrenchment on the understanding of action words and sentences, as opposed to abstract verbs, nouns, and adverbs^7,8,13,21–23^. Crucially, our results go beyond such findings by showing that similar effects can be triggered by whole-body physical activities and manifest in naturalistic discourse processes subsuming multiple verbal and non-verbal operations for the appraisal of textual information.

The above enhancement did not emerge for circumstantial information, which also remained unaffected in NTs. Note, in this sense, that circumstances are typically realized by non-obligatory adverbial or prepositional phrases, which renders them semantically and structurally independent from processes and verbs^19^. It is thus likely that the processing advantage triggered for action language did not spread over to information coded through these categories, as they hold no necessary semotactic and lexotactic links to action verbs. Indeed, a recent language embodiment model^6^ posits that verbal units which do not denote bodily actions more crucially rely on non-motor semantic circuits, whose presumable activation during our training protocol held no differential relation to either text type. Thus, discourse-level enhancements triggered by AVG training seem confined to action semantic fields, as opposed to the overall semantic structure of ATs. This further attests to the specificity of the link between motion-related mechanisms and action-language processing^24^.

Results from the subtraction analyses confirmed the above patterns, imposing even more stringent conditions. These statistical tests considered only the rate of change for each subject, thus revealing training-induced effects while controlling for performance in the Pre-T phase. Consequently, systematic stimulation of motor and motion-related sensory systems can selectively boost the appraisal of discourse-level action meanings irrespectively of baseline performance on a specific text type or word class.

So far, causal effects of sensorimotor-system activation on action language have been mainly obtained considering lists of isolated, randomly sequenced words (e.g., ref.^25^) or sentences (e.g., ref.^7^). Despite their major contributions, such findings are blind to the impact of sensorimotor-system entrenchment on discourse processing, and they cannot be *a priori* assumed to hold during processing of coherent and cohesive texts. Crucially, together with demonstrations that action-related information modulates somatosensory activations during story reading^9,10^ and becomes less critically manifest in spontaneous monologues of patients with motor disorders^11^, our results extend the field’s main empirical corpus, showing that embodied systems play a decisive role even when multiple cognitive mechanisms (e.g., lexical access, semantic integration, comprehension, recall) are jointly tapped via discourse-level tasks.

No less important is the finding that such enhancements can be induced by sustained real-life activities. This aligns with evidence that professional athletes are more efficient in processing verbs denoting discipline-specific actions^12,13^, with an important qualification: while the athletes’ advantage could merely be reflecting increased familiarity with those words, no such confound seems to undermine the present design. Therefore, we propose, action-language understanding can be selectively boosted by systematic, immersive training of motor and motion-related sensory systems.

Interestingly, the actions denoted by the ATs (e.g., running, cleaning) were not manipulated to match fine-grained features of the movements elicited by the AVGs (e.g., dancing, shaking the forearms). This observation suggests that activation of motor and motion-related sensory systems can impact action-language mechanisms at large. Previous studies have evinced fine-grained relations between action language and sensorimotor systems. For example, action verbs and sentences elicit somatotopic activity peaks in the motor/premotor cortices^24,26^, and manual movements are differentially or selectively affected by manual action verbs as opposed to foot- or mouth-related verbs^22,27,28^. However, effector-specific action-verbs also engage motor regions beyond somatotopic circuits^29–31^, and the spatial coordinates of their activation peaks do not align with probabilistically defined maps of the primary motor and premotor cortices^32^. Also, systematic repetition of one particular movement (e.g., moving beans towards the body) modulates processing of sentences involving *varied* directionally compatible action verbs^7^. Our findings further show that active engagement of movement and motion-perception mechanisms can have a coarse-grained impact on action semantics. This possibility, however, does not necessarily oppose the well-documented demonstration of effector-specific^6^, movement-direction-specific^33^, or gesture-speech congruency^34^ effects. Rather, we posit that such fine-grained associations may be *accompanied* by more widespread resonance throughout the motor system and the action stream.

Another crucial factor which likely contributed to the above pattern, and to our findings at large, is that our intervention also involved sustained visual attention to movements by the games’ characters. The same AVGs used in our protocol have been shown to favor processing of visuospatial motion patterns^15,16^, including enhancements of movement discrimination performance^16^. Such effects have been linked to increased engagement of the action stream (i.e., the magnocellular dorsal pathway)^35–37^, which may have also facilitated simulations of meanings evoked by verbs in the ATs. Compatibly, the mirror neuron system, comprising networks which fire similarly during movement execution and visualization, is implicated in action imagery^38^. Moreover, motor, somatosensory, and sensorimotor regions similarly recruited by actual and imagined movement are also significantly engaged during visual discrimination of movable body parts^39^. Hence, the effect triggered by AVGs in our study probably benefited from simultaneous boosting of motor and visuospatial mechanisms jointly stimulated by action execution and perception.

Of note, videogames can also enhance extralinguistic and non-motor functions in neurotypicals^40^ and, more particularly, short-term memory abilities in dyslexic children^16^. Indeed, the latter domain was significantly honed by our intervention, which could suggest that our findings were not strictly dependent on embodied language mechanisms. However, all reported effects (or lack thereof) remained unchanged after co-varying for short-term memory differences between the Pre-T and the Post-T phases. In line with evidence that action-semantic deficits in movement disorders are not explained by executive dysfunction^31,41^, these results suggest that the observed action-appraisal effect might be unmediated by extralinguistic functions. However, more systematic testing of additional domain-general skills (e.g., attention, inhibitory control) would be necessary to fully embrace this claim.

Furthermore, no differential effects between texts or verbal categories emerged in our mini-control-experiment, based on less dynamic, non-action videogames. Admittedly, the low sample size in this experiment raises reservations about its results. However, two independent studies using the same videogame intervention on samples of 10 or 11 dyslexic children^15,16^ showed that, although multiple cognitive skills (e.g., phonological decoding, repetition, reading, attention) were not improved after non-action-videogame training, all of them were enhanced after AVG training. Importantly, the same was true of movement discrimination performance^16^, a domain which, as stated above, is critically related to functioning of the magnocellular dorsal pathway, or action stream^35–37^. In light of these findings, the null effects in our mini-control-experiment seem attributable to low bodily and action perception demands rather than low statistical power proper. Indeed, results in our main experiment were replicated when analyses were performed on 18 out of 20 random subgroups of 10 subjects, matching the sample size of the mini-control-experiment. Tentatively, then, the selective enhancement in our main experiment seems to have been driven by the motoric and visuospatial aspects of the intervention protocol, as opposed to general features of videogaming at large. Nevertheless, this interpretation should be taken with caution until the mini-control-experiment is replicated with a more robust sample size.

In sum, whole-body AVG training seems capable of selectively enhancing the appraisal (including the comprehension) of action-related information during discourse-level processing. This link between two activities performed daily in real-life settings (videogame playing and story listening) offers an explicit empirical tie between embodied and situated accounts of cognition, opening new avenues for the development of the field.

### Limitations and suggestions for further research

The present study features some limitations. First, our linguistic assessment only considered receptive verbal skills. Further research could examine whether whole-body motor training can also have a differential impact on action-language production. Second, we only assessed changes immediately after training. By including additional post-training tests at successive time points, future studies could establish the durability of the observed effects. Third, as in previous studies based on similar materials^5^, our protocol was not aimed to disentangle the relative contribution of the multiple processes recruited for task completion. However, future elaborations of this study could assess such specificities by incorporating complementary tasks or by introducing additional factors in the design (e.g., manipulating memory demands by changing the temporal gap between text processing and questionnaire administration). It would also be useful to manipulate the degree of bodily motion on the part of the players and the characters, to ascertain the extent to which present findings were influenced by attention to visuospatial motion patterns. Finally, as noted in the Discussion, the small sample size of the mini-control-experiment weakens such complementary results and precludes direct comparisons with our main experiment. It would thus be critical to replicate such a control intervention with a larger sample size to more robustly establish whether our main finding was specifically driven by systematic bodily engagement as opposed to unspecific factors involved in videogaming at large.

Looking forward, our design could be adapted to explore links between other experiential and linguistic domains (e.g., between natural induction of affective states and processing of emotion-laden texts). This could further strengthen the empirical integration of embodied and situated frameworks in cognitive science. Finally, our findings may prompt ideas for clinical intervention. For example, it would be interesting to explore whether whole-body motor training can enhance everyday discourse processing in patients with action-related category specific deficits^42^. Moreover, action appraisal in naturalistic discourse is highly sensitive for classifying Parkinson’s disease patients from healthy subjects. Thus, in addition to broadening our understanding of language processing mechanisms, specific text-level measures could emerge as promising cognitive biomarkers of motor disorders^5^. Research in this direction would be critical to establish the translational relevance of the embodied framework at large.

## Conclusion

This is the first study to show that training of motor and motion-perception mechanisms through whole-body AVGs can selectively enhance the appraisal of actions evoked by naturalistic texts. Notably, since the latter did not match the actual movements from the participants or the videogame characters, it seems that sensorimotor grounding may operate in a coarse-grained fashion. Thus, embodied language mechanisms seem operative even during our dealings with the environment. Future applications and extensions of our design could offer critical data to further test the situated relevance of the embodiment framework.

## Electronic supplementary material

Supplementary Material

Action text 1 audio

Action text 2 audio

Neutral text 1 audio

Neutral text 2 audio

## References

[1] Gallese, V. & Lakoff, G. The brain’s concepts: the role of the sensory-motor system in conceptual knowledge. *Cogn Neuropsychol* **22**, 455–479, doi:714592738 (2005). 10.1080/02643290442000310 21038261

[2] Yang J (2014). Influences of motor contexts on the semantic processing of action-related language. Cog Aff Behav Neurosci.

[3] Hulme C, Snowling MJ (2014). The interface between spoken and written language: developmental disorders. Phil Trans R Soc Lond B Biol Sci.

[4] Xiao XY, Ho CSH (2014). Weaknesses in semantic, syntactic and oral language expression contribute to reading difficulties in Chinese dyslexic children. Dyslexia.

[5] García, A. M. *et al*. Parkinson’s disease compromises the appraisal of action meanings evoked by naturalistic texts. *Cortex* (accepted). 10.1016/j.cortex.2017.07.003 28764852

[6] Garcia AM, Ibanez A (2016). A touch with words: dynamic synergies between manual actions and language. Neurosci Biobehav Rev.

[7] Glenberg AM, Sato M, Cattaneo L (2008). Use-induced motor plasticity affects the processing of abstract and concrete language. Curr Biol.

[8] Locatelli M, Gatti R, Tettamanti M (2012). Training of manual actions improves language understanding of semantically related action sentences. Front Psychol.

[9] Desai RH, Choi W, Lai VT, Henderson JM (2016). Toward semantics in the wild: activation to manipulable nouns in naturalistic reading. J Neurosci.

[10] Speer NK, Zacks JM, Reynolds JR (2007). Human brain activity time-locked to narrative event boundaries. Psych Sci.

[11] Garcia AM (2016). How language flows when movements don’t: an automated analysis of spontaneous discourse in Parkinson’s disease. Brain Lang.

[12] Tomasino B, Guatto E, Rumiati RI, Fabbro F (2012). The role of volleyball expertise in motor simulation. Acta Psychol.

[13] Beilock SL, Lyons IM, Mattarella-Micke A, Nusbaum HC, Small SL (2008). Sports experience changes the neural processing of action language. Proc Nat Ac Sci USA.

[14] Tomasino B, Maieron M, Guatto E, Fabbro F, Rumiati RI (2013). How are the motor system activity and functional connectivity between the cognitive and sensorimotor systems modulated by athletic expertise?. Brain Res.

[15] Franceschini S (2013). Action video games make dyslexic children read better. Curr Biol.

[16] Gori, S., Seitz, A. R., Ronconi, L., Franceschini, S. & Facoetti, A. Multiple causal links between magnocellular-dorsal pathway deficit and developmental dyslexia. *Cereb Cortex*, 10.1093/cercor/bhv206 (2015). 10.1093/cercor/bhv206 PMC6317503 26400914

[17] Torgesen, J. K., Wagner, R. & Rashotte, C. TOWRE–2 Test of Word Reading Efficiency. *Austin, TX: Pro-Ed* (1999).

[18] Marinus E, Kohnen S, McArthur G (2013). Australian comparison data for the Test of Word Reading Efficiency (TOWRE). Aust J Learn Diff.

[19] Halliday, M. A. K. & Matthiessen, C. *Halliday’s Introduction to Functional Grammar*. Fourth ed, (Routledge, 2014).

[20] Davis CJ (2005). N-watch: a program for deriving neighborhood size and other psycholinguistic statistics. Behav Res Met.

[21] Boulenger V (2006). Cross-talk between language processes and overt motor behavior in the first 200 msec of processing. J Cog Neurosci.

[22] Dalla Volta R, Gianelli C, Campione GC, Gentilucci M (2009). Action word understanding and overt motor behavior. Exp Brain Res.

[23] Willems RM, Labruna L, D’Esposito M, Ivry R, Casasanto D (2011). A functional role for the motor system in language understanding: evidence from theta-burst transcranial magnetic stimulation. Psych Sci.

[24] Pulvermuller, F. Brain mechanisms linking language and action. *Nat Rev Neurosci* **6**, 576-582, doi:nrn1706 (2005). 10.1038/nrn1706 15959465

[25] Liuzzi G (2010). The involvement of the left motor cortex in learning of a novel action word lexicon. Curr Biol.

[26] Willems RM, Hagoort P, Casasanto D (2010). Body-specific representations of action verbs: neural evidence from right- and left-handers. Psych Sci.

[27] Buccino G (2005). Listening to action-related sentences modulates the activity of the motor system: a combined TMS and behavioral study. Brain Res.

[28] García AM, Ibáñez A (2016). Hands typing what hands do: action-semantic integration dynamics throughout written verb production. Cognition.

[29] Arevalo, A. L., Baldo, J. V. & Dronkers, N. F. What do brain lesions tell us about theories of embodied semantics and the human mirror neuron system? *Cortex* **48**, 242–254, doi:S0010-9452(10)00157-7 (2012). 10.1016/j.cortex.2010.06.001 PMC3615255 20621292

[30] Melloni M (2015). Cortical dynamics and subcortical signatures of motor-language coupling in Parkinson’s disease. Sci Rep.

[31] Ibanez A (2013). Motor-language coupling: direct evidence from early Parkinson’s disease and intracranial cortical recordings. Cortex.

[32] Kemmerer D, Gonzalez-Castillo J (2010). The Two-Level Theory of verb meaning: An approach to integrating the semantics of action with the mirror neuron system. Brain Lang.

[33] Kaschak MP, Borreggine KL (2008). Temporal dynamics of the action-sentence compatibility effect. Q J Exp Psychol (Hove).

[34] Wu YC, Coulson S (2015). Iconic gestures facilitate discourse comprehension in individuals with superior immediate memory for body configurations. Psych Sci.

[35] Talcott JB (2000). Dynamic sensory sensitivity and children’s word decoding skills. Proc Nat Ac Sci USA.

[36] Sperling AJ, Lu ZL, Manis FR, Seidenberg MS (2006). Motion-perception deficits and reading impairment: it’s the noise, not the motion. Psych Sci.

[37] Boets B (2013). Intact but less accessible phonetic representations in adults with dyslexia. Science.

[38] Binkofski F, Buccino G (2004). Motor functions of the Broca’s region. Brain Lang.

[39] Parsons LM (1995). Use of implicit motor imagery for visual shape discrimination as revealed by PET. Nature.

[40] Green CS, Bavelier D (2003). Action video game modifies visual selective attention. Nature.

[41] Bocanegra Y (2015). Syntax, action verbs, action semantics, and object semantics in Parkinson’s disease: dissociability, progression, and executive influences. Cortex.

[42] Capitani E, Laiacona M, Mahon B, Caramazza A (2003). What are the facts of semantic category-specific deficits? A critical review of the clinical evidence. Cog Neuropsych.

