# Supplementary material for: A moving story: Whole-body motor training selectively improves the appraisal of action meanings in naturalistic narratives
Source: Sci Rep. 2017 Oct 2;7:12538. doi: 10.1038/s41598-017-12928-w (PMC5624907; doi:10.1038/s41598-017-12928-w)
Supplement: Supplementary file 1 — Supplementary Material [file 41598_2017_12928_MOESM1_ESM.doc]

**A moving story: Whole-body motor training**

**selectively improves the appraisal of action meanings in naturalistic narratives**

Piergiorgio Trevisan, Lucas Sedeño, Agustina Birba,

Agustín Ibáñez, Adolfo M. García

**Supplementary material**

Main experiment

*Sample size estimation*

To determine the sample size required for our main experiment, we ran an estimation analysis in G*Power 3.1, a statistical software widely used in social and behavioral research^1^. Given our statistical design [factorial ANOVA with three factors (2x2x2)], we considered three parameters. First, we established alpha level of *p* = .05. Second, we considered an effect size of 0.60 (based on Cohen’s *f*). This large effect size was selected based on a previous study that found a robust improvement (*f* > 0.50) in reading abilities of dyslexic children after 12 hs of training with action video games^2^. Finally, we established a power of 0.8. This analysis showed that a sample size of 20 is adequate to reach the estimated effects.

*Replication on reduced subsamples*

To evaluate the influence of the sample size in our main results, we replicated the same factorial ANOVA for text appraisal results –i.e., a 2x2x2 design including the factors Text type (ATs and NTs), Information type (process related and circumstantial), and Time point (Pre-T and Post-T)− on 20 randomly selected subsamples. As reported in the main manuscript, 18 of these analyses yielded the same significant results reported with the whole sample in our main experiment, including the interaction between Text type, Information type, and Time point. For the latter, post-hoc tests revealed that the only significant improvement emerged in the appraisal of process-related information for ATs (*p*-value: *m* = .03, *SD =* .02), with no comparable effect for NTs. Details of each statistical model can be found in Table S1, and a graphical example of the results of one subsample is offered in Fig. S1.

Table S1. Statistical results for each of the 20 replications of the main experiment on random subsamples of size *n* = 10.

|  | Main effects | Tukey’s HSD post-hoc tests (interaction) |
| --- | --- | --- |
| 1 | Time point [*F* (1, 72) = 7.26, *p* = .008, *f* = 0.31]  (Post-T > Pre-T)* | Process-related information |
|  |  | ATs – Post-T > ATs – Pre-T : *p* = .01*  NTs – Post-T > NTs – Pre-T: *p* >.250  ATs – Pre-T vs NTs – Pre-T: *p* >.250 |
|  | Text type [*F* (1, 72) = 7.26, *p* = .008 , *f* = 0.31]  (ATs > NTs) * |  |
|  | Interaction between Text type, Information type, and Time point [*F* (1, 72) = 6.18, *p* = .015, *f* = 0.29] * | Circumstantial information |
|  |  | ATs – Post-T vs ATs – Pre-T: *p* >.250  NTs – Post-T vs NTs – Pre-T: *p* >.250  ATs – Pre-T vs NTs – Pre-T: *p* >.250 |
| 2 | Time point [*F* (1, 72) = 5.51, *p* = .04, *f* = 0.22]  (Post-T > Pre-T) * | Process-related information |
|  |  | ATs – Post-T > ATs – Pre-T : *p* = .02*  NTs – Post-T > NTs – Pre-T: *p* > .250  ATs – Pre-T vs NTs – Pre-T: *p* > .250 |
|  | Text type [*F* (1, 72) = 5.51, *p* = .04, *f* = 0.22]  (ATs > NTs) * |  |
|  | Interaction between Text type, Information type, and Time point [*F* (1, 72) = 6.81, *p* = .01, *f* = 0.29] * | Circumstantial information |
|  |  | ATs – Post-T vs ATs – Pre-T: *p* > .250  NTs – Post-T vs NTs – Pre-T: *p* > .250  ATs – Pre-T vs NTs – Pre-T: *p* >.250 |
| 3 | Time point [*F* (1, 72) = 12.10, *p* < .001, *f* = 0.40]  (Post-T > Pre-T) * | Process-related information |
|  |  | ATs – Post-T > ATs – Pre-T : *p* < .05*  NTs – Post-T > NTs – Pre-T: *p* > .250  ATs – Pre-T vs NTs – Pre-T: *p* > .250 |
|  | Text type [*F* (1, 72) = 1.34, *p* = .25, *f* = 0.13]  (ATs vs NTs) * |  |
|  | Interaction between Text type, Information type, and Time point [*F* (1, 72) = 6.31, *p* = .01 , *f* = 0.29] * | Circumstantial information |
|  |  | ATs – Post-T vs ATs – Pre-T: *p* > .250  NTs – Post-T vs NTs – Pre-T: *p* > .250  ATs – Pre-T vs NTs – Pre-T: *p* >.250 |
| 4 | Time point [*F* (1, 72) = 10.22, *p* = .002, *f* = 0.36]  (Post-T > Pre-T) * | Process-related information |
|  |  | ATs – Post-T > ATs – Pre-T : *p* = 01*  NTs – Post-T > NTs – Pre-T: *p* > .250  ATs – Pre-T vs NTs – Pre-T: *p* > .250 |
|  | Text type [*F* (1, 72) = 2.26, *p* = .13, *f* = 0.17]  (ATs vs NTs) |  |
|  | Interaction between Text type, Information type, and Time point [*F* (1, 72) = 14.14, *p* < .001, *f* = 0.43] * | Circumstantial information |
|  |  | ATs – Post-T vs ATs – Pre-T: *p* > .250  NTs – Post-T < NTs – Pre-T: *p* = .02*  ATs – Pre-T vs NTs – Pre-T: *p* >.250 |
| 5 | Time point [*F* (1, 72) = 10.32, *p* < .001, *f* = 0.36]  (Post-T > Pre-T) * | Process-related information |
|  |  | ATs – Post-T > ATs – Pre-T : *p* < .001*  NTs – Post-T > NTs – Pre-T: *p* > .250  ATs – Pre-T vs NTs – Pre-T: *p* > .250 |
|  | Text type [*F* (1, 72) = 2.25, *p* = .13, *f* = 0.17]  (ATs vs NTs) |  |
|  | Interaction between Text type, Information type, and Time point [*F* (1, 72) = 13.27, *p* < .001, *f* = 0.42] * | Circumstantial information |
|  |  | ATs – Post-T vs ATs – Pre-T: *p* > .250  NTs – Post-T vs NTs – Pre-T: *p* > .250  ATs – Pre-T vs NTs – Pre-T: *p* >.250 |
|  |  |  |
| 6 | Time point [*F* (1, 72) = 8.91, *p* = .003, *f* = 0.35]  (Post-T > Pre-T) * | Process-related information |
|  |  | ATs – Post-T > ATs – Pre-T : *p* = .05*  NTs – Post-T > NTs – Pre-T: *p* > .250  ATs – Pre-T vs NTs – Pre-T: *p* > .250 |
|  | Text type [*F* (1, 72) = 3.48, *p* = .07, *f* = 0.20]  (ATs vs NTs) |  |
|  | Interaction between Text type, Information type, and Time point [*F* (1, 72) = 8.91, *p* = .003, *f* = 0.35] * | Circumstantial information |
|  |  | ATs – Post-T vs ATs – Pre-T: *p* > .250  NTs – Post-T vs NTs – Pre-T: *p* > .250  ATs – Pre-T vs NTs – Pre-T: *p* >.250 |
| 7 | Time point [*F* (1, 72) = 5.98, *p* = .01, *f* = 0.27]  (Post-T > Pre-T) * | Process-related information |
|  |  | ATs – Post-T > ATs – Pre-T : *p* = .02*  NTs – Post-T > NTs – Pre-T: *p* > .250  ATs – Pre-T vs NTs – Pre-T: *p* > .250 |
|  | Text type [*F* (1, 72) = 1.27, *p* = .26, *f* = 0.10]  (ATs vs NTs) |  |
|  | Interaction between Text type, Information type, and Time point [*F* (1, 72) = 9.07, *p* = .003, *f* = 0.35] * | Circumstantial information |
|  |  | ATs – Post-T vs ATs – Pre-T: *p* > .250  NTs – Post-T vs NTs – Pre-T: *p* > .250  ATs – Pre-T vs NTs – Pre-T: *p* >.250 |
| 8 | Time point [*F* (1, 72) = 4.13, *p* = .04, *f* = 0.23]  (Post-T > Pre-T) * | Process-related information |
|  |  | ATs – Post-T > ATs – Pre-T : *p* = .04*  NTs – Post-T > NTs – Pre-T: *p* > .250  ATs – Pre-T vs NTs – Pre-T: *p* > .250 |
|  | Text type [*F* (1, 72) = 2.76, *p* = .10, *f* = 0.17]  (ATs vs NTs) |  |
|  | Interaction between Text type, Information type, and Time point [*F* (1, 72) = 9.87, *p* = .002, *f* = 0.37] * | Circumstantial information |
|  |  | ATs – Post-T vs ATs – Pre-T: *p* > .250  NTs – Post-T vs NTs – Pre-T: *p* > .250  ATs – Pre-T vs NTs – Pre-T: *p* >.250 |
| 9 | Time point [*F* (1, 72) = 5.20, *p* = .02, *f* = 0.25]  (Post-T > Pre-T) * | Process-related information |
|  |  | ATs – Post-T > ATs – Pre-T : *p* = .02*  NTs – Post-T > NTs – Pre-T: *p* > .250  ATs – Pre-T vs NTs – Pre-T: *p* > .250 |
|  | Text type [*F* (1, 72) = 5.20, *p* = .02, *f* = 0.25]  (ATs > NTs) * |  |
|  | Interaction between Text type, Information type, and Time point [*F* (1, 72) = 7.17, *p* = .009, *f* = 0.31] * | Circumstantial information |
|  |  | ATs – Post-T vs ATs – Pre-T: *p* > .250  NTs – Post-T vs NTs – Pre-T: *p* > .250  ATs – Pre-T vs NTs – Pre-T: *p* >.250 |
| 10 | Time point [*F* (1, 72) = 3.60, *p* = .06, *f* = 0.04]  (Post-T vs Pre-T) | Process-related information |
|  |  | ATs – Post-T > ATs – Pre-T : *p* = .02*  NTs – Post-T > NTs – Pre-T: *p* > .250  ATs – Pre-T vs NTs – Pre-T: *p* > .250 |
|  | Text type [*F* (1, 72) = 4.36, *p* = 0.04, *f* = 0.06]  (ATs > NTs) * |  |
|  | Interaction between Text type, Information type, and Time point [*F* (1, 72) = 9.23, *p* = .003, *f* = 0.11] * | Circumstantial information |
|  |  | ATs – Post-T vs ATs – Pre-T: *p* > .250  NTs – Post-T vs NTs – Pre-T: *p* > .250  ATs – Pre-T vs NTs – Pre-T: *p* >.250 |
| 11 | Time point [*F* (1, 72) = 8.72, *p* = .004, *f* = 0.35]  (Post-T > Pre-T) * | Process-related information |
|  |  | ATs – Post-T > ATs – Pre-T : *p* = .11  NTs – Post-T > NTs – Pre-T: *p* >.250  ATs – Pre-T vs NTs – Pre-T: *p* >.250 |
|  | Text type [*F* (1, 72) = 3.65, *p* = .06 , *f* = 0.23]  (ATs > NTs) |  |
|  | Interaction between Text type, Information type, and Time point [*F* (1, 72) = 3.02, *p* = .08, *f* = 0.20] | Circumstantial information |
|  |  | ATs – Post-T vs ATs – Pre-T: *p* >.250  NTs – Post-T vs NTs – Pre-T: *p* >.250  ATs – Pre-T vs NTs – Pre-T: *p* >.250 |
|  |  |  |
| 12 | Time point [*F* (1, 72) = 7.50, *p* = .007, *f* = 0.31]  (Post-T > Pre-T) * | Process-related information |
|  |  | ATs – Post-T > ATs – Pre-T : *p* = .02*  NTs – Post-T > NTs – Pre-T: *p* > .250  ATs – Pre-T vs NTs – Pre-T: *p* > .250 |
|  | Text type [*F* (1, 72) = 4.63, *p* = .03, *f* = 0.25]  (ATs > NTs) * |  |
|  | Interaction between Text type, Information type, and Time point [*F* (1, 72) = 6.47, *p* = .01, *f* = 0.29] * | Circumstantial information |
|  |  | ATs – Post-T vs ATs – Pre-T: *p* > .250  NTs – Post-T vs NTs – Pre-T: *p* > .250  ATs – Pre-T vs NTs – Pre-T: *p* >.250 |
| 13 | Time point [*F* (1, 72) = 4.92, *p* = .03, *f* = 0.25]  (Post-T > Pre-T) * | Process-related information |
|  |  | ATs – Post-T > ATs – Pre-T : *p* = .03*  NTs – Post-T > NTs – Pre-T: *p* > .250  ATs – Pre-T vs NTs – Pre-T: *p* > .250 |
|  | Text type [*F* (1, 72) = 4.10, *p* = .04, *f* = 0.22]  (ATs > NTs) * |  |
|  | Interaction between Text type, Information type, and Time point [*F* (1, 72) = 4.92, *p* = .03, *f* = 0.25] * | Circumstantial information |
|  |  | ATs – Post-T vs ATs – Pre-T: *p* > .250  NTs – Post-T vs NTs – Pre-T: *p* > .250  ATs – Pre-T vs NTs – Pre-T: *p* >.250 |
| 14 | Time point [*F* (1, 72) = 2.91, *p* = .09, *f* = 0.31]  (Post-T vs Pre-T) | Process-related information |
|  |  | ATs – Post-T > ATs – Pre-T : *p* = .01*  NTs – Post-T > NTs – Pre-T: *p* > .250  ATs – Pre-T vs NTs – Pre-T: *p* > .250 |
|  | Text type [*F* (1, 72) = 4.34, *p* = .04, *f* = 0.25]  (ATs > NTs) * |  |
|  | Interaction between Text type, Information type, and Time point [*F* (1, 72) = 9.18, *p* = .003, *f* = 0.35] * | Circumstantial information |
|  |  | ATs – Post-T vs ATs – Pre-T: *p* > .250  NTs – Post-T vs NTs – Pre-T: *p* > .250  ATs – Pre-T vs NTs – Pre-T: *p* >.250 |
| 15 | Time point [*F* (1, 72) = 11.40, *p* = .001, *f* = 0.38]  (Post-T > Pre-T) * | Process-related information |
|  |  | ATs – Post-T > ATs – Pre-T : *p* = .02*  NTs – Post-T > NTs – Pre-T: *p* > .250  ATs – Pre-T vs NTs – Pre-T: *p* > .250 |
|  | Text type [*F* (1, 72) = 6.67, *p* = .01, *f* = 0.29]  (ATs > NTs) * |  |
|  | Interaction between Text type, Information type, and Time point [*F* (1, 72) = 6.67, *p* = .01, *f* = 0.29] * | Circumstantial information |
|  |  | ATs – Post-T vs ATs – Pre-T: *p* > .250  NTs – Post-T vs NTs – Pre-T: *p* > .250  ATs – Pre-T vs NTs – Pre-T: *p* >.250 |
| 16 | Time point [*F* (1, 72) = 7.68, *p* = .007, *f* = 0.31]  (Post-T > Pre-T) * | Process-related information |
|  |  | ATs – Post-T > ATs – Pre-T : *p* = .03*  NTs – Post-T > NTs – Pre-T: *p* > .250  ATs – Pre-T vs NTs – Pre-T: *p* > .250 |
|  | Text type [*F* (1, 72) = 1.35, *p* = .24, *f* = 0.10]  (ATs vs NTs) |  |
|  | Interaction between Text type, Information type, and Time point [*F* (1, 72) = 5.00, *p* = .02, *f* = 0.25] * | Circumstantial information |
|  |  | ATs – Post-T vs ATs – Pre-T: *p* > .250  NTs – Post-T vs NTs – Pre-T: *p* > .250  ATs – Pre-T vs NTs – Pre-T: *p* >.250 |
| 17 | Time point [*F* (1, 72) = 8.16, *p* = .005, *f* = 0.33]  (Post-T > Pre-T) * | Process-related information |
|  |  | ATs – Post-T > ATs – Pre-T : *p* = .09  NTs – Post-T > NTs – Pre-T: *p* > .250  ATs – Pre-T vs NTs – Pre-T: *p* > .250 |
|  | Text type [*F* (1, 72) = 10.67, *p* = .001, *f* = 0.36]  (ATs > NTs) * |  |
|  | Interaction between Text type, Information type, and Time point [*F* (1, 72) = 2.04, *p* = .15, *f* = 0.14] | Circumstantial information |
|  |  | ATs – Post-T vs ATs – Pre-T: *p* > .250  NTs – Post-T vs NTs – Pre-T: *p* > .250  ATs – Pre-T vs NTs – Pre-T: *p* >.250 |
|  |  |  |
| 18 | Time point [*F* (1, 72) = 11.14, *p* = .001, *f* = 0.38]  (Post-T > Pre-T) * | Process-related information |
|  |  | ATs – Post-T > ATs – Pre-T : *p* = .02*  NTs – Post-T > NTs – Pre-T: *p* > .250  ATs – Pre-T vs NTs – Pre-T: *p* > .250 |
|  | Text type [*F* (1, 72) = 2.63, *p* = .10, *f* = 0.17]  (ATs vs NTs) |  |
|  | Interaction between Text type, Information type, and Time point [*F* (1, 72) = 4.81, *p* = .03, *f* = 0.25] * | Circumstantial information |
|  |  | ATs – Post-T vs ATs – Pre-T: *p* > .250  NTs – Post-T vs NTs – Pre-T: *p* > .250  ATs – Pre-T vs NTs – Pre-T: *p* >.250 |
| 19 | Time point [*F* (1, 72) = 15.22, *p* < .001, *f* = 0.45]  (Post-T > Pre-T) * | Process-related information |
|  |  | ATs – Post-T > ATs – Pre-T : *p* = .01*  NTs – Post-T > NTs – Pre-T: *p* > .250  ATs – Pre-T vs NTs – Pre-T: *p* > .250 |
|  | Text type [*F* (1, 72) = 1.86, *p* = .17, *f* = 0.14]  (ATs vs NTs) |  |
|  | Interaction between Text type, Information type, and Time point [*F* (1, 72) = 7.45, *p* = .007, *f* = 0.31] * | Circumstantial information |
|  |  | ATs – Post-T vs ATs – Pre-T: *p* > .250  NTs – Post-T vs NTs – Pre-T: *p* > .250  ATs – Pre-T vs NTs – Pre-T: *p* >.250 |
| 20 | Time point [*F* (1, 72) = 11.04, *p* = .004, *f* = 0.38]  (Post-T > Pre-T) * | Process-related information |
|  |  | ATs – Post-T > ATs – Pre-T : *p* = .002*  NTs – Post-T > NTs – Pre-T: *p* > .250  ATs – Pre-T vs NTs – Pre-T: *p* > .250 |
|  | Text type [*F* (1, 72) = 1.94, *p* = .16, *f* = 0.14]  (ATs vs NTs) |  |
|  | Interaction between Text type, Information type, and Time point [*F* (1, 72) = 8.37, *p* = .005, *f* = 0.33] * | Circumstantial information |
|  |  | ATs – Post-T vs ATs – Pre-T: *p* > .250  NTs – Post-T vs NTs – Pre-T: *p* > .250  ATs – Pre-T vs NTs – Pre-T: *p* >.250 |
| Asterisks (*) indicate significant differences. | | |

**
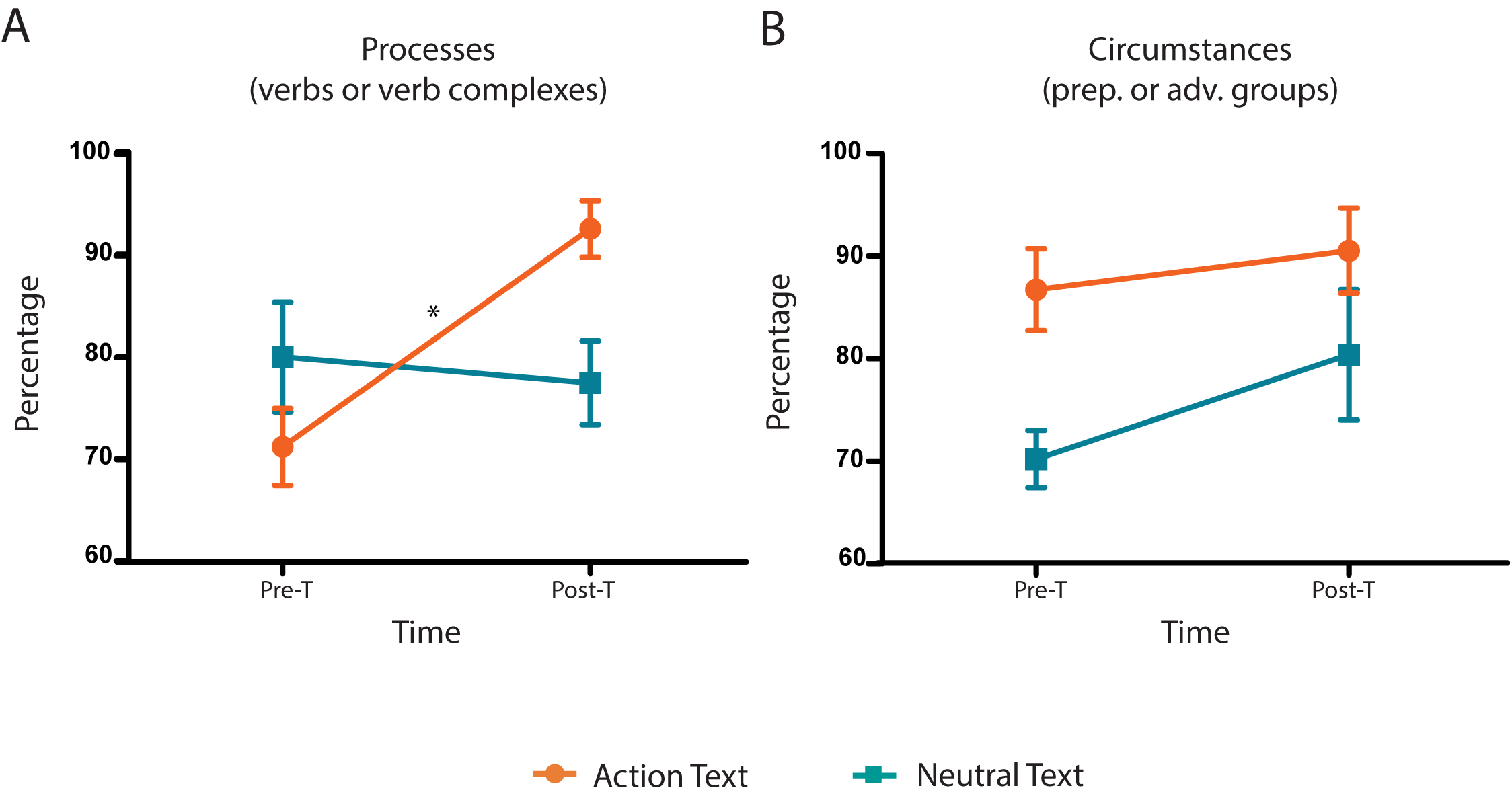
**

**Figure S1.** Text appraisal results from a group of 10 random AVG participants. **A.** The appraisal of process-related information (realized by verbs or verb groups) increased significantly only for action texts after AVG training. **B.** The appraisal of circumstantial information (realized by prepositional or adverbial groups) was unaffected by AVG training in both action and neutral texts. Values on the Y-axes indicate percentage scores. Asterisks (*) indicate significant differences. Pre-T: pre-test phase; Post-T: post-test phase.

Mini-control-experiment

*Participants*

The mini-control-experiment involved 10 dyslexic English-speaking children (1 female), all of whom met the study’s inclusion criteria (see main text, section ‘Participants’). Six of them were right-handed, and none had visual, auditory, or motor impairments. The group had a mean age of 10.6 years (*SD* = 2.1) and an average of 4.3 years of education (*SD* = 2). Their mild dyslexic profile was confirmed by their performance on word reading (percentile of the sample’s mean = 4.89) and phonemic decoding (percentile of the sample’s mean = 8.75), as assessed through the Sight Words and Phonemic Decoding subtests of TOWRE 2^3^, respectively. Thus, in both subtests, the sample fell in the lowest 30% of the Australian population, based on age-specific norms^4^.

This mini-control group was matched in gender (χ^2^ = 2.13, *p* = .144), age [*F*(1, 28) = 1.71, *p* = .201], years of education [*F*(1, 28) = 1.75, *p* = .196], and handedness (χ^2^ = 2.32, *p* = .126) with our main experimental group.

*Non-action video games: further details*

The non-action games we used are all part of a unified storyline, in which a character called Rayman has to accomplish various tasks to earn his freedom. Each task corresponds to a minigame set in a specific location (e.g., the Far West, the sea), generally involving some sort of confrontation between Rayman and a group of bunnies. The selected games are quite mechanical; they can be performed exclusively with simple wrist/hand responses and they elicit no leg movements. For example, the games “Bunnies can’t Fly” and “Bunnies don’t Give Gifts” could easily be played with eyes closed, as they only required the children to repeatedly perform a simple manual reaction for a very short time.

*Results*

*Text appraisal: ANOVA results*

There were no main effects of Text type [*F*(1, 72) = 0.04, *p* > .250, *f* = 0.00], Information type [*F*(1, 72) = 1.06, *p* > .250, *f* = 0.01], or Time point [*F*(1, 72) = 1.36, *p* > .250, *f* = 0.14]. Crucially, however, unlike what was observed in the main experiment, the interaction among these three factors was not significant [*F*(1, 72) = 1.06, *p* > .250, *f* = 0.10] –Fig. S2, Panels A and B. Such results were corroborated by a subtraction analysis between Post-T and Pre-T performance [*F*(1, 36) = 1.27, *p* > .250, *f* = 0.17] –Fig. S2, Panel C.


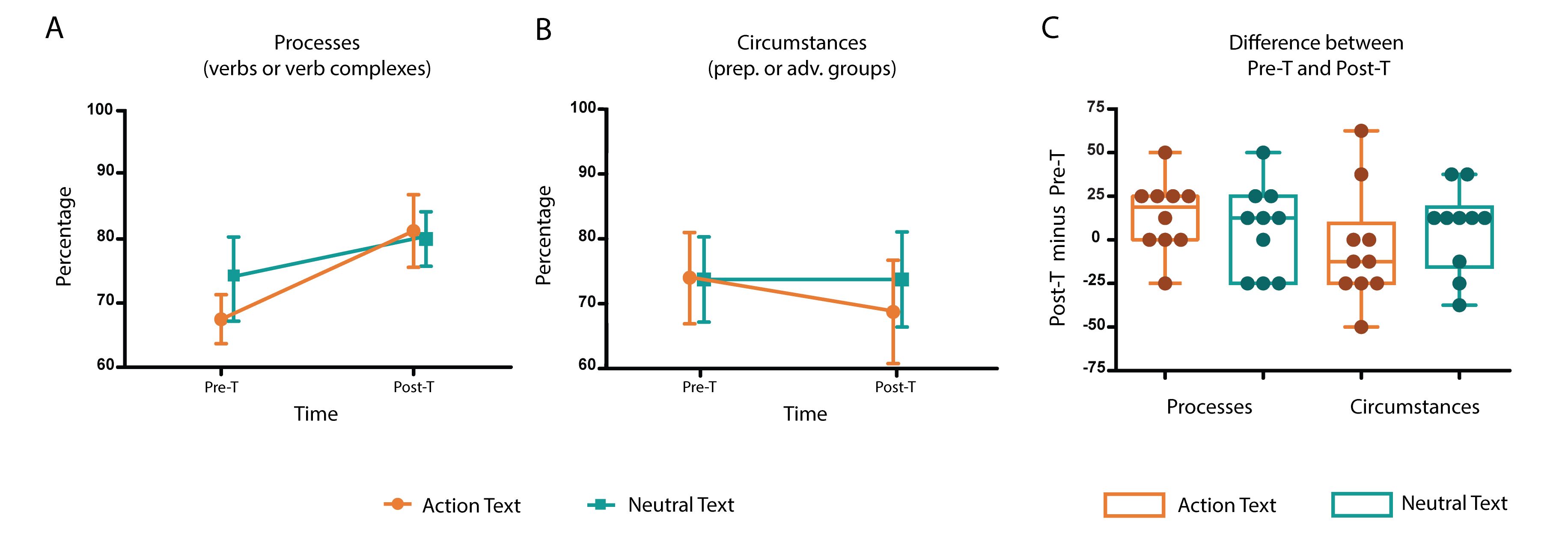


**Figure S2.** Text appraisal results from the mini-control-experiment. **A.** The appraisal of process-related information (realized by verbs or verb groups) was unaffected by non-action-videogame training in both action and neutral texts. **B.** The appraisal of circumstantial information (realized by prepositional or adverbial groups) was unaffected by non-action-videogame training in both action and neutral texts. **C.** Subtraction analyses corroborated the finding that non-action-videogame training selectively boosted the appraisal of process-related information for action texts. Values on the Y-axes indicate percentage scores. Pre-T: pre-test phase; Post-T: post-test phase.

*Short-term memory: ANOVA results*

A comparison between short-term memory scores in Pre-T (*M* = 2.9, *SD* = 0.87) and Post-T (*M* = 2.3, *SD* = 1.06) showed no significant training effects [*F*(1, 18) = 1.90, *p* = .184, *f* = 0.31].

**Supplementary references**

1 Faul, F., Erdfelder, E., Lang, A. G. & Buchner, A. G*Power 3: a flexible statistical power analysis program for the social, behavioral, and biomedical sciences. *Behav Res Met* **39**, 175-191 (2007).

2 Franceschini, S. *et al.* Action video games make dyslexic children read better. *Curr Biol* **23**, 462-466, doi:10.1016/j.cub.2013.01.044 (2013).

3 Torgesen, J. K., Wagner, R. & Rashotte, C. *TOWRE–2 Test of Word Reading Efficiency*. Austin, TX: Pro-Ed (1999).

4 Marinus, E., Kohnen, S. & McArthur, G. Australian comparison data for the Test of Word Reading Efficiency (TOWRE). *Aust J Learn Diff* **18**, 199-212, doi:10.1080/19404158.2013.852981 (2013).

**Questionnaires**

**Questionnaire for action text 1**

| 1 | What did Donald  do with his bag? | 1. | He opened it |  |
| --- | --- | --- | --- | --- |
|  |  | 2. | He inspected it |  |
|  |  | 3. | He threw it away |  |
|  |  | 4. | He closed it |  |
|  |  | 5. | I don’t remember |  |
| 2 | Where did Donald  look for his money? | 1. | In his car |  |
|  |  | 2. | At the airport |  |
|  |  | 3. | In his bag |  |
|  |  | 4. | In his drawer |  |
|  |  | 5. | I don’t remember |  |
| 3 | What did Donald  do with great exultation? | 1. | He sang |  |
|  |  | 2. | He jumped |  |
|  |  | 3. | He ran |  |
|  |  | 4. | He slept |  |
|  |  | 5. | I don’t remember |  |
| 4 | How did Donald  count the money Tommy gave him? | 1. | In great exultation |  |
|  |  | 2. | With sadness |  |
|  |  | 3. | With some joy |  |
|  |  | 4. | With his eyes closed |  |
|  |  | 5. | I don’t remember |  |
| 5 | How did Donald  go to his friend Tommy’s? | 1. | Driving |  |
|  |  | 2. | Walking |  |
|  |  | 3. | Running |  |
|  |  | 4. | Swimming |  |
|  |  | 5. | I don’t remember |  |
| 6 | Where did Donald go soon after receiving the money from Tommy? | 1. | To New Zealand |  |
|  |  | 2. | To the post office |  |
|  |  | 3. | To the supermarket |  |
|  |  | 4. | To the newspaper office |  |
|  |  | 5. | I don’t remember |  |
| 7 | What was Tommy doing  when Donald rang the bell? | 1. | Cleaning |  |
|  |  | 2. | Working |  |
|  |  | 3. | Sleeping |  |
|  |  | 4. | Flying |  |
|  |  | 5. | I don’t remember |  |

| 8 | Where did Donald put his money  while he was speaking to the receptionist? | 1. | By the window |  |
| --- | --- | --- | --- | --- |
|  |  | 2. | On a chair |  |
|  |  | 3. | On the table |  |
|  |  | 4. | In the lift |  |
|  |  | 5. | I don’t remember |  |
| 9 | What happened  to Donald’s money bag? | 1. | He lent it to a friend |  |
|  |  | 2. | He lost it |  |
|  |  | 3. | He burnt it |  |
|  |  | 4. | He forgot it |  |
|  |  | 5. | I don’t remember |  |
| 10 | Where did the receptionist put the ad? | 1. | On a poster |  |
|  |  | 2. | In a website |  |
|  |  | 3. | In the newspaper |  |
|  |  | 4. | In the garden |  |
|  |  | 5. | I don’t remember |  |
| 11 | Why did a man  go to see Donald? | 1. | He had found the money bag |  |
|  |  | 2. | He had not seen the money bag |  |
|  |  | 3. | He wanted to have fun |  |
|  |  | 4. | He had seen the money bag |  |
|  |  | 5. | I don’t remember |  |
| 12 | A man went… | 1. | To Donald’s office |  |
|  |  | 2. | To Donald’s parents |  |
|  |  | 3. | To University |  |
|  |  | 4. | To Donald’s house |  |
|  |  | 5. | I don’t remember |  |
| 13 | What did Donald  and the man do later? | 1. | They saw a movie together |  |
|  |  | 2. | They had breakfast together |  |
|  |  | 3. | They had dinner together |  |
|  |  | 4. | They cut a tree together |  |
|  |  | 5. | I don’t remember |  |
| 14 | Where did Donald  take the man, later? | 1. | To Tasmania |  |
|  |  | 2. | To a bar |  |
|  |  | 3. | To a restaurant |  |
|  |  | 4. | To a Mac Donald’s |  |
|  |  | 5. | I don’t remember |  |

| 15 | What was Donald doing  while thinking what to do? | 1. | Working in the garden |  |
| --- | --- | --- | --- | --- |
|  |  | 2. | Walking in the room |  |
|  |  | 3. | Running in the park |  |
|  |  | 4. | Building a house |  |
|  |  | 5. | I don’t remember |  |
| 16 | How was Donald moving, while thinking? | 1. | Back and forth |  |
|  |  | 2. | Very fast |  |
|  |  | 3. | Vertically |  |
|  |  | 4. | In circles |  |
|  |  | 5. | I don’t remember |  |

**Questionnaire for neutral text 1**

| 1 | How did the inhabitants treat each other where Poppy lived? | 1. | They were indifferent to each other |  |
| --- | --- | --- | --- | --- |
|  |  | 2. | They supported each other |  |
|  |  | 3. | They fought against each other |  |
|  |  | 4. | They liked each other |  |
|  |  | 5. | I don’t remember |  |
| 2 | Where did Poppy live? | 1. | In a big city |  |
|  |  | 2. | On the moon |  |
|  |  | 3. | In a town |  |
|  |  | 4. | In a village |  |
|  |  | 5. | I don’t remember |  |
| 3 | Why did Poppy start a journey? | 1. | To see his relatives |  |
|  |  | 2. | To buy some chocolate |  |
|  |  | 3. | To exercise for a marathon |  |
|  |  | 4. | To find out the truth about something |  |
|  |  | 5. | I don’t remember |  |
| 4 | How did Poppy feel in his place? | 1. | Happy |  |
|  |  | 2. | Sad |  |
|  |  | 3. | Quite OK |  |
|  |  | 4. | Always very sick |  |
|  |  | 5. | I don’t remember |  |
| 5 | During his journey, Poppy… | 1. | Never walked |  |
|  |  | 2. | Never spoke |  |
|  |  | 3. | Never felt sick |  |
|  |  | 4. | Never felt sad |  |
|  |  | 5. | I don’t remember |  |

| 6 | Where did Poppy go on his journey? | 1. | To a nearby river |  |
| --- | --- | --- | --- | --- |
|  |  | 2. | To a very far town |  |
|  |  | 3. | To Europe |  |
|  |  | 4. | To a nearby village |  |
|  |  | 5. | I don’t remember |  |
| 7 | What was the problem with the lie  that a stranger had told? | 1. | Poppy’s friends were not sure about it |  |
|  |  | 2. | Poppy’s friends had believed in it |  |
|  |  | 3. | Poppy’s friends beat the stranger |  |
|  |  | 4. | Poppy’s friends rejected it |  |
|  |  |  |  |  |
| 8 | How did Poppy think  about the girl’s words? | 1. | In great curiosity |  |
|  |  | 2. | With some interest |  |
|  |  | 3. | Disgustingly |  |
|  |  | 4. | With fun |  |
|  |  | 5. | I don’t remember |  |
| 9 | What did Poppy do  with all the chocolate  he collected during his journey? | 1. | He brought it back with him |  |
|  |  | 2. | He ate it |  |
|  |  | 3. | He cooked it |  |
|  |  | 4. | He sold it |  |
|  |  | 5. | I don’t remember |  |
| 10 | Where were Poppy’s friends  when that stranger came? | 1. | On a mountain |  |
|  |  | 2. | On the roof of a church |  |
|  |  | 3. | In a city nearby |  |
|  |  | 4. | In their home place |  |
|  |  | 5. | I don’t remember |  |
| 11 | How did Poppy spend his time  when he went back from his journey? | 1. | Sleeping |  |
|  |  | 2. | Teaching |  |
|  |  | 3. | Reading |  |
|  |  | 4. | Playing football |  |
|  |  | 5. | I don’t remember |  |
| 12 | When did Poppy finally found out  the truth about chocolate? | 1. | Before arriving at his journey’s destination |  |
|  |  | 2. | A couple of years later |  |
|  |  | 3. | After arriving at his journey’s destination |  |
|  |  | 4. | During school exams |  |
|  |  | 5. | I don’t remember |  |
| 13 | What did Poppy’s friends think about chocolate at the beginning of the text? | 1. | They loved it |  |
|  |  | 2. | They hated the smell of it |  |
|  |  | 3. | They were curious about it |  |
|  |  | 4. | They were frightened of it |  |
|  |  | 5. | I don’t remember |  |
| 14 | How much time did Poppy spend with his friends, after the journey? | 1. | Very little time |  |
|  |  | 2. | Seven days and night |  |
|  |  | 3. | Some days |  |
|  |  | 4. | A lot of time |  |
|  |  | 5. | I don’t remember |  |
| 15 | A girl, at the beginning of the text, said that the inhabitants would sick if | 1. | They had touch chocolate |  |
|  |  | 2. | They had seen chocolate on TV |  |
|  |  | 3. | They had eaten chocolate |  |
|  |  | 4. | They had smelt chocolate |  |
|  |  | 5. | I don’t remember |  |
| 16 | When did Poppy decide  to start his journey? | 1. | After hearing the girl’s words |  |
|  |  | 2. | Before hearing the girl’s words |  |
|  |  | 3. | Yesterday |  |
|  |  | 4. | One year later |  |
|  |  | 5. | I don’t remember |  |

**Questionnaire for action text 2**

| 1 | Where was Bobby at 8 am? | 1. | At home |  |
| --- | --- | --- | --- | --- |
|  |  | 2. | At lunch |  |
|  |  | 3. | At the cinema |  |
|  |  | 4. | At work |  |
|  |  | 5. | I don’t remember |  |
| 2 | What was Bobby worried about  at the beginning of the text? | 1. | Having locked his car |  |
|  |  | 2. | Having taken his lunch |  |
|  |  | 3. | Having locked his house |  |
|  |  | 4. | Having fed his cat |  |
|  |  | 5. | I don’t remember |  |
| 3 | Where was Bobby’s bag? | 1. | In a drawer |  |
|  |  | 2. | In a cupboard |  |
|  |  | 3. | Under the table |  |
|  |  | 4. | In the fridge |  |
|  |  | 5. | I do not remember |  |
| 4 | How did Bobby  communicate with Pablo? | 1. | He called him through skype |  |
|  |  | 2. | He sent him a text message |  |
|  |  | 3. | He wrote him an email |  |
|  |  | 4. | He called him on the phone |  |
|  |  | 5. | I don’t remember |  |
| 5 | In what mood did Bobby  communicate with Pablo? | 1. | With some anxiety |  |
|  |  | 2. | Very quietly |  |
|  |  | 3. | Very joyfully |  |
|  |  | 4. | In big panic |  |
|  |  | 5. | I don’t remember |  |
| 6 | What was Pablo doing  when Bobby called him? | 1. | Having a bath |  |
|  |  | 2. | Cleaning |  |
|  |  | 3. | Washing the dishes |  |
|  |  | 4. | Flying to Melbourne |  |
|  |  | 5. | I don’t remember |  |
| 7 | Where did Pablo live? | 1. | Near Bobby’s house |  |
|  |  | 2. | Near Bobby’s workplace |  |
|  |  | 3. | Near his sister’s house |  |
|  |  | 4. | In another city |  |
|  |  | 5. | I don’t remember |  |
| 8 | Where did Pablo go  shortly afterwards? | 1. | He ran to to Bobby’s workplace |  |
|  |  | 2. | He walked to his wife’s house |  |
|  |  | 3. | He went to Bobby’s house |  |
|  |  | 4. | He drove to the supermarket |  |
|  |  | 5. | I don’t remember |  |
| 9 | What did Pablo do for Bobby? | 1. | Shouted the windows |  |
|  |  | 2. | Repaired the TV |  |
|  |  | 3. | Locked the door |  |
|  |  | 4. | Cooked dinner |  |
|  |  | 5. | I don’t remember |  |
| 10 | What did Pablo ask his wife to do? | 1. | Call Bobby |  |
|  |  | 2. | Send Bobby a message |  |
|  |  | 3. | Go to Bobby’s office |  |
|  |  | 4. | Cook some pizza |  |
|  |  | 5. | I don’t remember |  |
| 11 | Before 6 pm, Bobby…. | 1. | Came back from work |  |
|  |  | 2. | Had a coffee |  |
|  |  | 3. | Went to the swimming pool |  |
|  |  | 4. | Cooked a pizza |  |
|  |  | 5. | I don’t remember |  |
| 12 | Where did Bobby and Pablo meet,  after work? | 1. | At Pablo’s house |  |
|  |  | 2. | At Bobby’s house |  |
|  |  | 3. | At the cinema |  |
|  |  | 4. | In the city centre |  |
|  |  | 5. | I don |  |
| 13 | How did Pablo and Bobby  celebrate their friendship? | 1. | They went to the movies |  |
|  |  | 2. | They spent some days together |  |
|  |  | 3. | They had dinner together |  |
|  |  | 4. | They had a drink |  |
|  |  | 5. | I don’t remember |  |

| 14 | How did Pablo eat the cake? | 1. | With disgust |  |
| --- | --- | --- | --- | --- |
|  |  | 2. | With eagerness |  |
|  |  | 3. | Against his will |  |
|  |  | 4. | With joy |  |
|  |  | 5. | I don’t remember |  |
| 15 | Where did Pablo and Bobby  spend the night? | 1. | At the stadium |  |
|  |  | 2. | At a restaurant |  |
|  |  | 3. | On an island |  |
|  |  | 4. | At home |  |
|  |  | 5. | I don’t remember |  |
| 16 | What time did they go back? | 1. | Very late |  |
|  |  | 2. | At 10 pm |  |
|  |  | 3. | Very early |  |
|  |  | 4. | Ten minutes later |  |
|  |  | 5. | I don’t remember |  |

**Questionnaire for neutral text 2**

| 1 | Where did Lucy live? | 1. | In a big city |  |
| --- | --- | --- | --- | --- |
|  |  | 2. | In a small city |  |
|  |  | 3. | In a village |  |
|  |  | 4. | On a mountain |  |
|  |  | 5. | I don’t remember |  |
| 2 | What did people find hard to do,  where Lucy lived? | 1. | To have dinner together |  |
|  |  | 2. | To go hunting together |  |
|  |  | 3. | To talk to each other |  |
|  |  | 4. | To help each other |  |
|  |  | 5. | I don’t remember |  |
| 3 | How did Lucy feel in the place  in which she lived? | 1. | She liked it |  |
|  |  | 2. | She hated it |  |
|  |  | 3. | She tried to escape many times |  |
|  |  | 4. | She felt sad |  |
|  |  | 5. | I don’t remember |  |
| 4 | What did Lucy’s schoolmates  think of the place they lived in? | 1. | They did not like it |  |
|  |  | 2. | They were indifferent to it |  |
|  |  | 3. | They hated it |  |
|  |  | 4. | They liked it |  |
|  |  | 5. | I do not remember |  |
| 5 | Lucy thought  of her schoolmates’ words… | 1. | With fun |  |
|  |  | 2. | With some interest |  |
|  |  | 3. | With no interest |  |
|  |  | 4. | Without understanding them |  |
|  |  | 5. | I don’t remember |  |
| 6 | Where did Lucy go  after talking to her schoolmates? | 1. | To a restaurant |  |
|  |  | 2. | To Betta’s workplace |  |
|  |  | 3. | To Betta’s house |  |
|  |  | 4. | To Melbourne |  |
|  |  | 5. | I don’t remember |  |
| 7 | How did Lucy go there? | 1. | She rode a bike |  |
|  |  | 2. | She took a bus |  |
|  |  | 3. | She flew with an airplane |  |
|  |  | 4. | She walked |  |
|  |  | 5. | I don’t remember |  |
| 8 | Why did Lucy go to see Betta? | 1. | To have some fun |  |
|  |  | 2. | To go out together |  |
|  |  | 3. | To find out something |  |
|  |  | 4. | To go on vacation |  |
|  |  | 5. | I don’t remember |  |
| 9 | What did Lucy find hard to do? | 1. | To believe in her schoolmates |  |
|  |  | 2. | To study |  |
|  |  | 3. | To go to school |  |
|  |  | 4. | To admire the fireworks |  |
|  |  | 5. | I don’t remember |  |
| 10 | What did Lucy and Betta do  to find out the truth? | 1. | They drank something |  |
|  |  | 2. | They ate something |  |
|  |  | 3. | They went to the gym |  |
|  |  | 4. | They watched something |  |
|  |  | 5. | I don’t remember |  |
| 11 | When did Lucy and Betta  try the potion together? | 1. | Some time later |  |
|  |  | 2. | On the same day |  |
|  |  | 3. | One month later |  |
|  |  | 4. | On the next day |  |
|  |  | 5. | I don’t remember |  |
| 12 | When did Lucy and Betta  find out the truth? | 1. | When they talked to their friend Armando |  |
|  |  | 2. | Before trying the potion |  |
|  |  | 3. | When they went on holiday |  |
|  |  | 4. | After trying the potion |  |
|  |  | 5. | I don’t remember |  |
| 13 | Lucy and Betta were very curious… | 1. | After trying the potion |  |
|  |  | 2. | When the football match started |  |
|  |  | 3. | When they met their schoolmates |  |
|  |  | 4. | Before trying the potion |  |
|  |  | 5. | I don’t remember |  |
| 14 | When did Lucy pretend  to be very happy? | 1. | Before trying the potion |  |
|  |  | 2. | After trying the potion |  |
|  |  | 3. | Some months ago |  |
|  |  | 4. | Last week |  |
|  |  | 5. | I don’t remember |  |
| 15 | Lucy and Betta discovered  that Lucy’s schoolmates | 1. | Had made fun of her |  |
|  |  | 2. | Were right |  |
|  |  | 3. | Had gone on a trip |  |
|  |  | 4. | Could cook very well |  |
|  |  | 5. | I don’t remember |  |
| 16 | Where was Lucy when she pretended  to be very happy? | 1. | At home |  |
|  |  | 2. | At the gym with Betta |  |
|  |  | 3. | At school |  |
|  |  | 4. | At the bus stop |  |
|  |  | 5. | I don’t remember |  |
